# Treatment of severe refractory immune neutropenia with ruxolitinib: Two cases

**DOI:** 10.70962/jhi.20250131

**Published:** 2025-09-08

**Authors:** Richard Schwarzkopf, Andrew A.Y. Chen, Stephanie Quon, Julia Varghese, Janet Roberts, Richard K. Wood, Luke Y.C. Chen

**Affiliations:** 1 Department of Psychology and Neuroscience, Dalhousie University, Halifax, Canada; 2 Department of Biochemistry and Molecular Biology, Dalhousie University, Halifax, Canada; 3 Faculty of Medicine, University of British Columbia, Vancouver, Canada; 4 Division of Hematology, University of British Columbia, Vancouver, Canada; 5 Division of Rheumatology, Department of Medicine, Dalhousie University, Halifax, Canada; 6 Department of Pathology, Dalhousie University, Halifax, Canada; 7 Division of Hematology, Dalhousie University, Halifax, Canada

## Abstract

Two adult patients with severe immune neutropenia refractory to standard therapies achieved durable remission following treatment with the JAK inhibitor ruxolitinib. This report highlights the potential of JAK–STAT pathway inhibition for managing G-CSF and steroid-refractory immune-mediated neutropenia.

## Teaser

We describe two cases of severe refractory immune neutropenia: One with T-large granular lymphocyte leukemia and one with chronic idiopathic neutropenia. The JAK inhibitor ruxoltinib restored normal neutrophil counts and was well tolerated in both cases. Further investigation of ruxolitinib as a treatment for immune neutropenia is warranted.

Severe acquired neutropenia in adults is a significant therapeutic challenge. Two common causes are chronic idiopathic neutropenia (CIN) and T-large granular lymphocyte leukemia (T-LGLL) (1). Neutropenia in T-LGLL is mediated largely by dysregulation of the Janus kinase (JAK)/signal transducer and activator of transcription (STAT) pathway. This leads to excessive inflammatory cytokines, such as interferon γ and the interleukin (IL)-1 superfamily, increased number of cytotoxic granules, and increased apoptosis of mature granulocytes (2). The mechanisms underlying CIN are less well-defined but are thought to be related to a pro-inflammatory bone marrow milieu with increased Fas antigen expression and excess inflammatory cytokines, such as tumor necrosis factor, leading to granulocyte hypoplasia, maturation arrest, and apoptosis of granulocyte progenitors (3).

First-line treatment for those with T-LGLL and severe neutropenia is typically immunosuppression with either methotrexate, cyclosporine, or cyclophosphamide. The JAK–STAT inhibitor ruxolitinib has recently emerged as a treatment option in patients with T cell lymphoproliferative leukemia refractory to standard therapy, including T-LGLL (2). In contrast, treatment options for severe CIN (SCIN), defined as neutrophils chronically <0.5 × 10^9^/liter, are sparse. A small proportion of patients will respond to corticosteroids or the immunosuppressive therapies used for T-LGLL. The only proven therapy is granulocyte colony-stimulating factor (G-CSF), which often causes fever and arthralgias, particularly in younger patients, and must be continued chronically (4). To our knowledge, no effective therapies have been reported for patients with SCIN refractory to G-CSF.

**Figure 1.**
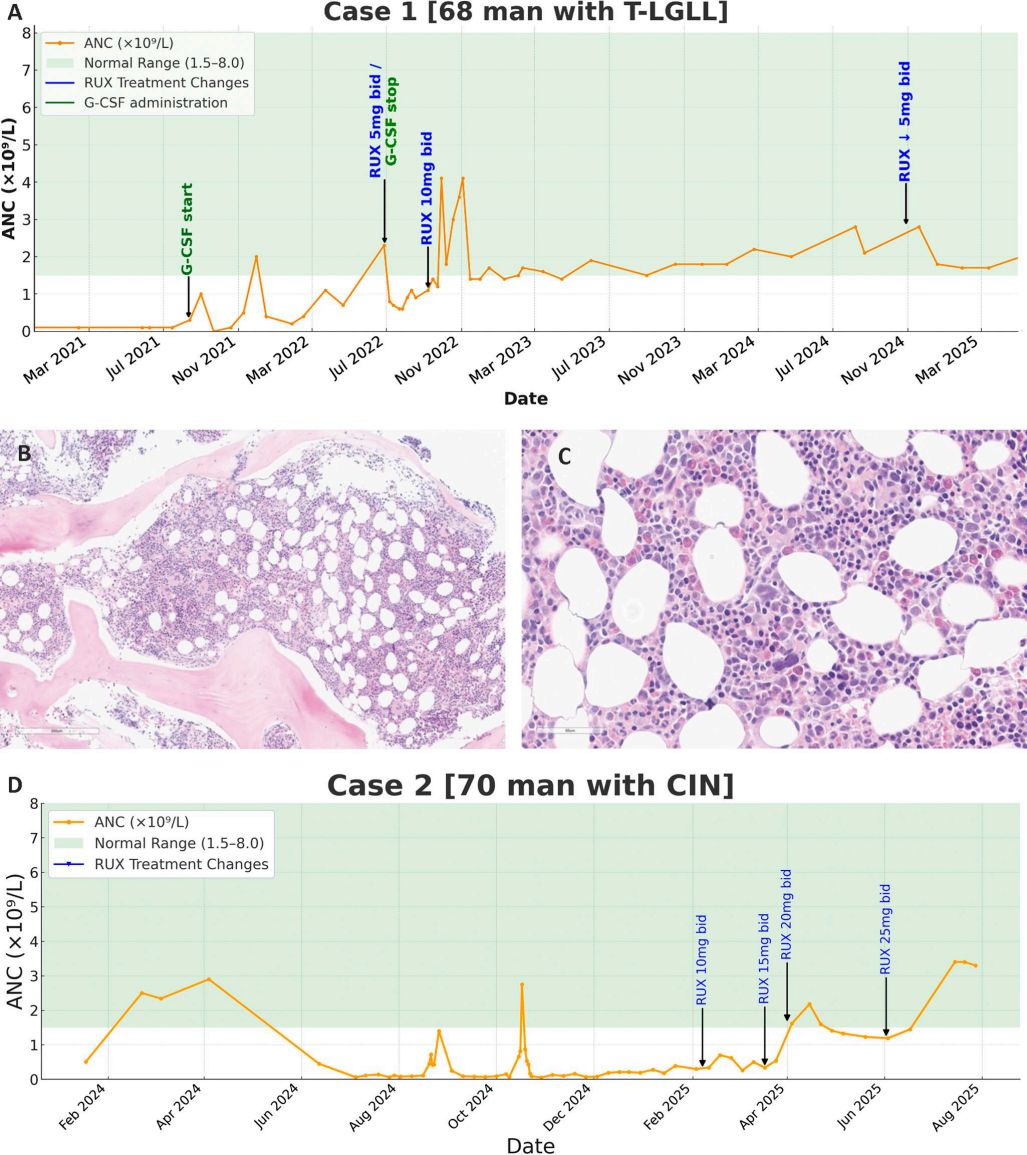
**Neutrophil trends and bone marrow findings in two patients with immune neutropenia treated with ruxolitinib. (A and D)** Neutrophil counts for case 1 (A) and case 2 (D) over time, with shaded areas indicating the normal range (1.5–8.0 × 10^9^/liter) and annotated arrows marking ruxolitinib (RUX) dosing changes. **(B and C)** Case 2 bone marrow biopsy (B and C) shows slightly hypercellular bone marrow with trilineage hematopoiesis and a relative decrease in mature neutrophils. Panels show hematoxylin and eosin–stained sections at 50× (B) and 400× (C) magnification.

We report two cases of severe refractory neutropenia in adult males, one with T-LGLL refractory to numerous immunosuppressive agents and another with CIN refractory to both immunosuppressives and G-CSF. Both patients achieved a durable response on therapy with ruxolitinib. The first case is a 68-year-old white man who presented with febrile neutropenia and T-LGLL in 2009. On examination, he had a fever of 39°C, with no focus of infection and no lymphadenopathy or splenomegaly. His white blood cell count was 4.3 × 10^9^/liter with an absolute neutrophil count (ANC) of 0.0 × 10^9^/liter (ref. 2.0–7.0). He had hemoglobin of 131 g/liter (ref. 135–170), platelets of 331 × 10^9^/liter (ref 150–400), creatinine of 156 µmol/liter (ref 49–93), and an estimated glomerular filtration rate (eGFR) of 39 ml/min/1.73 m^2^. Ferritin was 187 µg/liter (ref 20–300); vitamin B12 was 278 pmol/liter (ref >150); and calcium, C-reactive protein (CRP), and lactate dehydrogenase (LDH) were all within normal limits. Serum protein electrophoresis showed mild polyclonal hypergammaglobulinemia, gamma globulins 14.5 g/liter (ref 6–13) with IgG 17.4 g/liter (6.3–14.5), and normal IgA and IgM; anti-neutrophil antibody (ANA), anti-neutrophils cytoplasmic antibodies (ANCA), rheumatoid factor (RF), and direct antiglobulin test (DAT) were negative, as were HIV and hepatitis B and C serology. Computed tomography (CT) of the chest, abdomen, and pelvis revealed mild splenomegaly 14 × 10.5 × 5.5 cm. Bone marrow biopsy revealed normoblastic erythropoiesis, left-shifted granulopoiesis with 1% blasts, 10% involvement by an unusual CD8^+^ T cell population with dim CD5 expression, and 50% total marrow cellularity. Clonality was positive by T cell receptor PCR. He did not respond to G-CSF or corticosteroids. He underwent splenectomy with diagnostic and therapeutic intent; the pathology showed a mildly enlarged spleen of 495 g with no evidence of lymphoproliferative leukemia or other abnormality. After splenectomy, his neutrophils transiently went above 1.0 × 10^9^/liter for a month before falling back to <0.3 × 10^9^/liter chronically. He was given immunosuppressives, including oral cyclophosphamide 100 mg per os (po) daily for 3 mo, cyclosporine 100 mg po bis in die (bid) for 3 mo, methotrexate 20 mg po weekly for 5 mo, azathioprine 100 mg po daily for 3 mo, alone and in combination with corticosteroids, with no response. From 2009 to 2022, he had two to four episodes of febrile neutropenia per year, requiring visits to the emergency department and hospitalization. G-CSF 480 μg daily for 4–7 days would transiently raise his neutrophils to >1.0 × 10^9^/liter when he had infections, but regular G-CSF twice weekly was ineffective as maintenance therapy. He was started on ruxolitinib 5 mg bid in June 2022, and his dose was increased to 10 mg bid in September 2022. On ruxolitinib, he had an excellent clinical and hematological response, maintaining a neutrophil count >1.5 × 10^9^/liter (Fig. 1 A). Subjectively, his energy levels and quality of life improved, and he has not experienced any severe infections or need for antibiotics.

The second case is a 77-year-old white man with SCIN and subsequent diagnosis of rheumatoid arthritis. In the fall of 2023, he developed fatigue, decreased appetite, and recurrent sinus infections. He had increased joint pain and stiffness that was typical of inflammatory arthritis throughout the ensuing months, as well as a psoriasiform rash that resolved with 0.1% Betaderm. Initial physical examination revealed no hepatosplenomegaly, lymphadenopathy, or palpable joint effusions. Antiviral prophylaxis with valacyclovir was used to treat his history of mouth ulcers and herpes labialis outbreaks. Laboratory tests showed persistent neutropenia with ANCs consistently below 0.5 × 10^9^/liter (Fig. 1 D), along with mild normocytic anemia (hemoglobin 120 g/liter). Initial immunologic workup showed a positive ANA (via bead assay) with positive anti-RNP at low titer of 1.0 antibody index (normal ≤ 0.9), and elevated RF (37.5 Iµ/ml). ANCA and DAT were negative. Renal function tests showed an eGFR of 52 ml/min/1.73 m^2^ and a creatinine of 106 μmol/liter (ref 49–93). Inflammatory markers included an undetectable CRP and a ferritin level of 224 μg/liter. Calcium and liver enzyme were normal; serum protein electrophoresis showed mild polyclonal hypergammaglobulinemia 17.4 g/liter (ref 7.5–14.5) and IgG was mildly elevated 16.5 g/liter (6.5–14.2) as was IgA 4.0 g/liter (0.95–3.6) with normal IgM 2.5 g/liter. CT of the head, neck, chest, and abdomen showed mild sinusitis and no evidence of lymphadenopathy, splenomegaly, or malignancy. The peripheral blood smear showed marked neutropenia with no overt dysplasia. The bone marrow aspirate and biopsy showed slightly hypercellular marrow spaces with a relative decrease in mature neutrophils, 12% of nucleated cells (Fig. 1, B and C). Flow cytometry on the aspirate specimen showed a slight reversal of the CD4:CD8 T cell ratio with no phenotypically distinct T cell population. He then developed worsening joint pain, with synovitis noted on clinical examination. Given high titer anti-cyclic citrullinated peptide antibodies (152 µ/ml), he was diagnosed with seropositive rheumatoid arthritis (RA). Treatment with prednisone (25 mg daily tapered over 10 wk) and hydroxychloroquine 400 mg daily led to symptomatic improvement of his arthritis, but his neutropenia persisted. He was treated with methotrexate 20 mg weekly for 2 mo, azathioprine 100 mg po daily for 2 mo, mycophenolate mofetil 500 mg po bid for 6 wk, G-CSF 480 μg twice weekly, cyclophosphamide 100 mg po daily for 1 mo, and cyclosporine 100 mg po bid for 2 mo, all with low-dose corticosteroids with no response. The hydroxychloroquine was primarily for his arthritis, and the methotrexate was intended to treat both the RA and the SCIN. Although he initially had transient responses to prednisone and G-CSF with brief improvement in his neutrophil counts, these were short-lived. Over this 10-mo period, he had several infections requiring antibiotics and two admissions for febrile neutropenia.

In February 2025, he began ruxolitinib initially dosed at 10 mg bid. This was titrated over several weeks to 15 mg bid and eventually 20 mg bid, alongside low-dose prednisone 10 mg po daily with good tolerability. No improvement in his neutrophils was seen at the lower doses of ruxolitinib, but at a dose of 20 mg bid, his neutrophils rose to >1.0 × 10^9^/liter and have been maintained at that level for several months, and his prednisone was tapered off over 3 mo (Fig. 1 D).

These two cases demonstrate that JAK inhibition can be effective in treating severe immune-mediated neutropenia. The clonal expansion of cytotoxic CD8^+^ T cells and ongoing stimulation of the JAK–STAT pathway play key roles in the development of T-LGLL. A recent study of 21 patients with LGL leukemia demonstrated an impressive overall response rate of 86% (18/21), including 3 complete responses and 15 partial responses. 11 of the patients were refractory to cyclophosphamide, methotrexate, and cyclosporine, the standard immunosuppressive therapies of T-LGLL. Nine of these patients responded to ruxolitinib (2). To this existing literature, we add our experience of a patient with highly refractory LGLL who achieved a sustained response with ruxolitinib.

We further postulated that there may be overlap in the pathophysiology of CIN and T-LGLL. Our patient with severe, symptomatic CIN did not respond to low-dose ruxolitinib but had an excellent sustained response while on ruxolitinib 20 mg bid. To our knowledge, this is the first report of JAK inhibition as a treatment for CIN. Drug repurposing is an essential strategy for orphan diseases, wherein there is little incentive for industry-sponsored pharmaceutical research (5). Ruxolitinib was developed primarily for use in myelofibrosis, wherein it can worsen cytopenias, including neutropenia in some patients, and thus may not be an intuitive choice for patients with CIN. However, in drawing from experience in T-LGLL, we believe further exploration of JAK–STAT dysregulation as a driver of CIN is warranted.
